# Optimization of Degradation Conditions with PRG, a Polysaccharide from *Phellinus ribis*, by RSM and the Neuroprotective Activity in PC12 Cells Damaged by Aβ_25–35_

**DOI:** 10.3390/molecules24163010

**Published:** 2019-08-20

**Authors:** Pei Yang, Juan Jin, Qian Liu, Dongmei Ma, Jia Li, Yongqing Zhang, Yuhong Liu

**Affiliations:** 1 School of Pharmaceutical Sciences, Shandong University of Traditional Chinese Medicine, Jinan 250355, China; 2 School of foreign language of Shandong University of Traditional Chinese Medicine, Jinan 250355, China

**Keywords:** polysaccharide, *Phellinus ribis*, degradation, neuroprotevtive activity, amyloid-β, mitochondrial membrane permeability, cytochrome C

## Abstract

In the previous work, we found PRG, a polysaccharide from *Phellinus ribis*, exhibited neurotrophic activity. To obtain an active structural unit with lower molecular weight, PRG was degraded to prepare the degraded PRG (DPRG) using ascorbic acid and H_2_O_2_. The aim of the paper was to obtain DPRG by optimizing the degradation conditions using response surface methodology (RSM) and to study its protective effects of PC12 cells induced by Aβ_25–35_. The optimum conditions were as follows; the concentration of H_2_O_2_-Vc was 17 mM and degradation temperature was 50 °C; when degradation time was 1.6 h, the experimental response value of PC12 cell viability was 83.4 ± 0.15%, which was in accordance with the predicted value (83.5%). We also studied the protective effects of DPRG against the Aβ_25–35_-induced neurotoxicity and explored the underlying mechanism. The results showed that treatment with DPRG could attenuate PC12 cells death. The mechanism was relative to the inhibition of cell apoptosis by increasing the MMP level and decreasing the protein expression of cytochrome C (Cytc) in PC12 cells. In conclusion, DPRG with lower molecular weight was obtained successfully. It possessed neuroprotective properties and might be a candidate for neurodegenerative disease treatment.

## 1. Introduction

Alzheimer’s disease (AD) is a deadly chronic neurodegenerative disorder in the world, which contributes to memory loss and cognitive decline in the elderly [1,2]. It is estimated that there are millions of people suffering AD over the world [3]. Many studies reported that one of the causes of AD was the accumulation of amyloid-β (Aβ) fibrillar deposition [4]. Aβ_25–35_, the toxic peptide fragment of the full-length Aβ peptide, could induce a direct toxic effect on the neurons, leading to apoptosis of neurons [5,6]. In fact, as it has been recently shown, the toxicity of peptide fragments, such as Aβ, depended on its concentration and aggregation status and in certain conditions such as subtoxic levels or monomeric form could be protective for neurons by inducing the release of neurotrophic factors [7,8,9]. After screening, we finally determined that Aβ_25–35_ was incubated for one week to induce its aggregation and the cell damage rate was ~50% when the concentration was 30 μM. Therefore, in the present paper, the inhibition of Aβ-induced neuronal apoptosis may provide a promising approach for the prevention and treatment of neurodegenerative diseases especially AD.

Apoptosis occurs throughout the life of multicellular organisms, which can remove excess and damaged cells in the body and maintain the stability of tissues and organs. Eukaryotic cells mediate apoptosis mainly through the death receptor-mediated external apoptotic pathway, the internal mitochondrial pathway, and the endoplasmic reticulum stress pathway that have been concerned in recent years. There are four main types of protein molecules involved in these apoptotic processes: caspases, adapter proteins, Bcl-2, and inhibitor of apoptosis proteins (IAPs). The mitochondrial pathway, which activates caspase through the apoptosis activating factor released by mitochondria, is one of the most important pathways, which is composed of Bcl3-containing Bcl-2 family members (Bid, Bad, Bim, Harikari, Noxa, etc.) and other Bcl-2 family members (Bax subfamily members Bax, Bak, etc.) that bind to the outer surfaces of the mitochondria or exist in the cytosol. Through the interaction of two Bcl-2 proteins, the latter is oligomerized and inserted into the mitochondrial membrane, causing changes in mitochondrial membrane permeability (MMP) and loss of transmembrane potential, releasing Cytc and other proteins. The release of Cytc is a critical step in the mitochondrial apoptotic pathway [10]. This paper demonstrates that the inhibition of degraded PRG (DPRG) on the apoptosis of PC12 cells was associated with mitochondrial membrane potential and Cytc.

**Figure 1:**
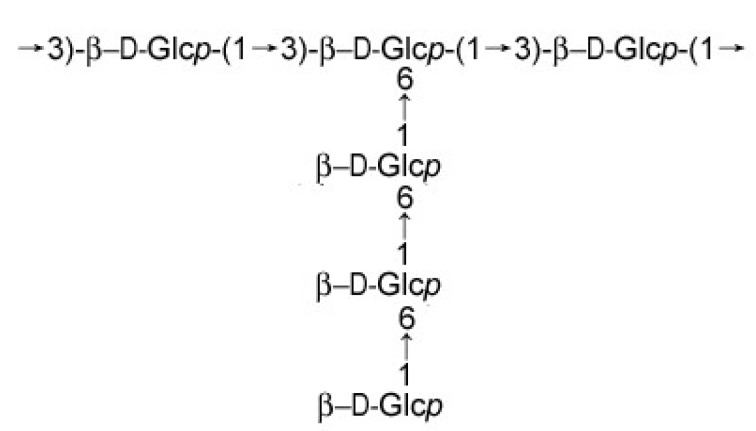
Schematic of the repeating unit of PRG.

At present, there is no effective treatment for AD. More and more evidence indicates that natural polysaccharides extracted from animals and plants possess a range of biological activities, for example, they exhibit antioxidant properties [11], and are used for neurodegenerative diseases [12]. In the previous work, a polysaccharide named PRG was isolated from the medicinal fungus *Phellinus ribis*. It is a β-d-glucan containing a (1→3)-linked backbone, with a branch of three (1→6)-linked glucoses substituting at the C-6 position every three residues along the main chain (Figure 1). PRG significantly promoted the neurite outgrowth of the nerve growth factor-mediated PC12 cells, exhibiting the neurotrophic activity [13]. However, the biological activities of the polysaccharide are usually limited by the high molecular weight [14]. It is necessary to get active structural unit of polysaccharide with lower molecular weight to increase water solubility or accelerate absorption and transport. So the present paper is concerned with the degradation of PRG, the optimization of degradation conditions by response surface methodology (RSM) and the protective effects of the degraded PRG on Aβ_25–35_-induced damage in PC12 cells.

## 2. Results and Discussion

### 2.1. Optimization of the Degradation Conditions by RSM

RSM, an effective statistical method and mathematical techniques in optimizing complex systems, is usually used to obtain the optimal parameters by constructing mathematical models and analysis of regression and variance [15,16,17]. By using RSM, the number of experimental trials was reduced and the interactions between independent variables were illustrated [18,19]. As one of the most popular forms of RSM, BBD has been widely used in most experiments [20]. In this study, BBD was utilized to assess the influence of three individual factors including concentration of H_2_O_2_-Vc (A), degradation temperature (B), degradation time (C), and their interaction effects on the viability of PC12 cells damaged by Aβ_25–35_. The response experimental values under different treatment conditions are shown in Table 1. By applying multiple regression analysis on the experimental data, the equation was as follows (Equation (1)):

Y = 82.77 + 3.08A + 0.96B + 0.85C − 0.25AB + 0.18AC + 0.17BC − 3.89A^2^ − 4.19B^2^ − 3.59C^2^ (1)

while Y is the response variable of PC12 cell viability, A, B, and C are three independent variables as shown in Table 2. The ANOVA study for response surface quadratic model was used to evaluate the significance of the regression equation and the adequacy of the model, and the results are shown in Table 3.

The *p*-value (*p* < 0.05) and F-value determine the significant degree of each coefficient. The *p*-value becomes smaller suggesting the corresponding variables become more effective [21]. In our results, the *p*-value (*p* < 0.01) and the *F*-value (617.18) demonstrated that model term was significant. The *p*-value of “lack-of-fit” was 0.8704, which showed that the “lack-of-fit” was not significant relative to the pure error [22,23]. The values of R^2^ (0.9987) and adjusted R^2^ (0.9971) were closed to 1, indicating the high efficacy and general availability of the equation. In conclusion, the regression model for viability of PC12 cells was a good prediction of the experimental results.

**Figure 2:**
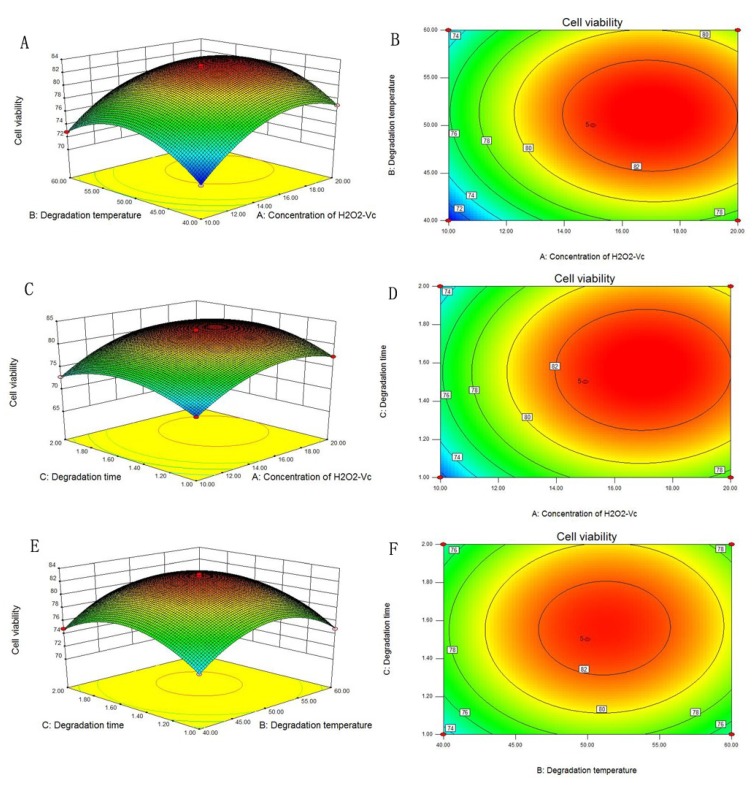
Response surface plots (**A**,**C**,**E**) and contour plots (**B**,**D**,**F**) of PC12 cells viability: the interaction between concentration of H_2_O_2_-Vc and degradation temperature (**A**,**B**), the interaction between concentration of H_2_O_2_-Vc and degradation time (**C**,**D**), and the interaction between degradation temperature and time (**E**,**F**).

The three-dimensional (3D) response surface and two-dimensional (2D) contour plots, which showed the response over a region of independent variables and their interactions, are indicated in Figure 2. The results in Figure 2A,B revealed the effects of H_2_O_2_-Vc concentration and degradation temperature to the response value, showing that PC12 cells viability increased upon increasing concentration of H_2_O_2_-Vc and temperature. A maximal response value was obtained when the concentration of H_2_O_2_-Vc was 16.98 mM and temperature was 51 °C. Subsequently, the viability of PC12 cells declined with the enhancement of degradation temperature and concentration of H_2_O_2_-Vc. Figure 2C,D described the effects of degradation temperature and time, the results demonstrated that the cell viability increased in first and decreased at last with increasing of the two factors. Simultaneously, Figure 2E,F showed the effect of concentration of H_2_O_2_-Vc and degradation time on response value, exhibiting a similar tendency compared with that in Figure 2C,D.

Based on the analysis results, the optimal conditions of concentration of H_2_O_2_-Vc, degradation temperature and time were found to be 16.98 mM, 51 °C and 1.57 h, respectively. Nevertheless, considering the operability and convenience in the production, the experimental parameters can be modified: the concentration of H_2_O_2_-Vc was 17 mM and the degradation temperature was 50 °C, while the degradation time was 1.6 h. Under these conditions, the experimental cell viability was 83.4 ± 0.15%, which was very close to the predicted result (83.5%). These results suggested the regression model was accurate and appropriate for the prediction of polysaccharide degradation. Under the optimal condition, the degraded PRG was further purified and DPRG was obtained.

### 2.2. Characterization of DPRG

DPRG appeared as a slight yellow power. HPGPC analysis calculated the average molecular weight was 3.06 kDa. The retention time of DPRG was later than PRG, indicating that the DPRG was degraded successfully by H_2_O_2_-Vc. TLC of hydrolyzate and GC of the alditol acetates were conducted to investigate the monosaccharide composition of DPRG by comparing with standard monosaccharides. The results showed that DPRG consists only of glucose and did not contain glucuronic acid, which was the same as PRG.

**Figure 3:**
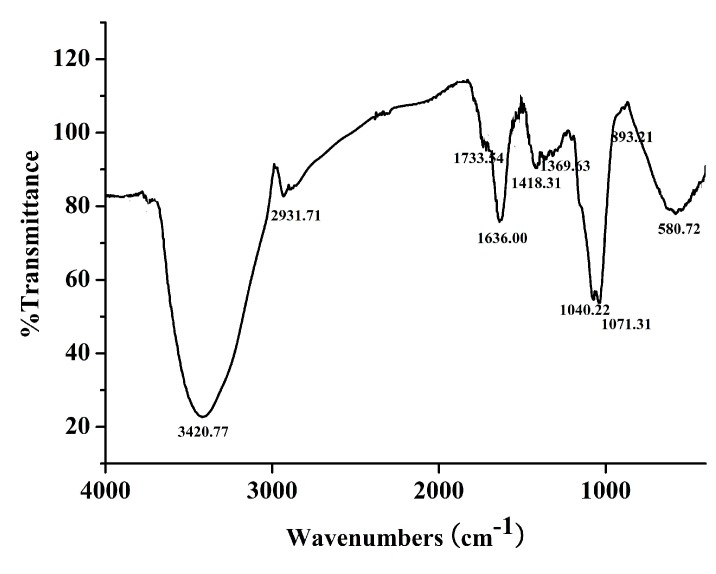
FTIR spectrum of degraded PRG (DPRG).

The FTIR (Figure 3) showed a strong absorption at 3420.77 cm^−1^, indicating the hydroxyl stretching vibration of the polysaccharide. The bands at 1040.22 cm^−1^, 1071.31 cm^−1^ suggested the presence of C–O–C and C–O–H in the pyran structure. The signal at 2931.71 cm^−1^ was due to C–H stretching vibration and a characteristic absorption at 893.21cm^−1^ was indicative of β-configuration in DPRG [24].

### 2.3. Effect of DPRG on Cell Viability in Aβ_25–35_-Damaged PC12 Cells

PC12 cells, a sort of tumor cell line isolated from rats, are widely used as model in the research of the mechanisms involved in neurotoxicity, neuroprotection and neuronal differentiation because of its performance in growth characteristics, which was similar to neurons, like cell aggregation and the emergence of fibrous ridges [25,26].

Aβ_25–35_, the toxic peptide fragment of Aβ protein, was known to induce neuronal cell death and result in apoptosis or necrosis of PC12 cells in AD models under proper aggregation status and concentration [27,28,29]. Therefore, we adopted Aβ_25–35_-damaged PC12 cells as the AD model in vitro to observe the neuroprotective effect of PRG and DPRG by MTT assay, which is based on the conversion of MTT to formazan crystals by mitochondrial dehydrogenases [30].

**Figure 4:**
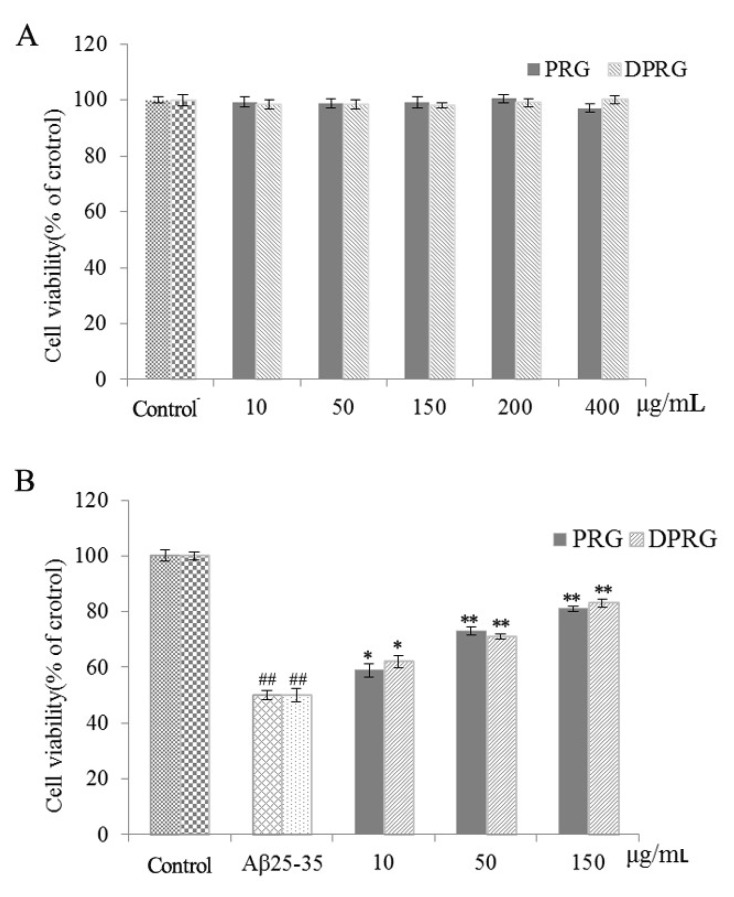
Effects of PRG and DPRG on the viability of PC12 cells in (**A**) basal or (**B**) Aβ_25–35_-induced conditions. Data shown are representative of three independent experiments. The reported values are the mean ± SD, *n* = 3. ^##^ *p* < 0.01 compared to control group; * *p* < 0.05, ** *p* < 0.01 compared to model group.

First of all, we studied the effect of PRG and DPRG on PC12 cells without Aβ_25–35_ incubation. The results showed both PRG and DPRG had no significant cytotoxicity in the range of 10-400 μg/mL (Figure 4A). However, normal PC12 cells incubated with Aβ_25–35_ for a period of time could induce cell death [28]. As shown in Figure 4B, the cell viability of model group was decreased to 52.5% after 24 h incubation with Aβ_25–35_ compared with that of control group (*p* < 0.01). Pretreatment with different concentrations of PRG and DPRG could increase the cell viability. As a result, the active structural unit of PRG with lower molecular weight was obtained, which had significant neurotrophic activity like PRG. So in the following experiments, DPRG was used to explore the mechanisms of the neuroprotective effect.

### 2.4. Effect of DPRG on Aβ_25–35_-Induced Apoptosis in PC 12 Cells

**Figure 5:**
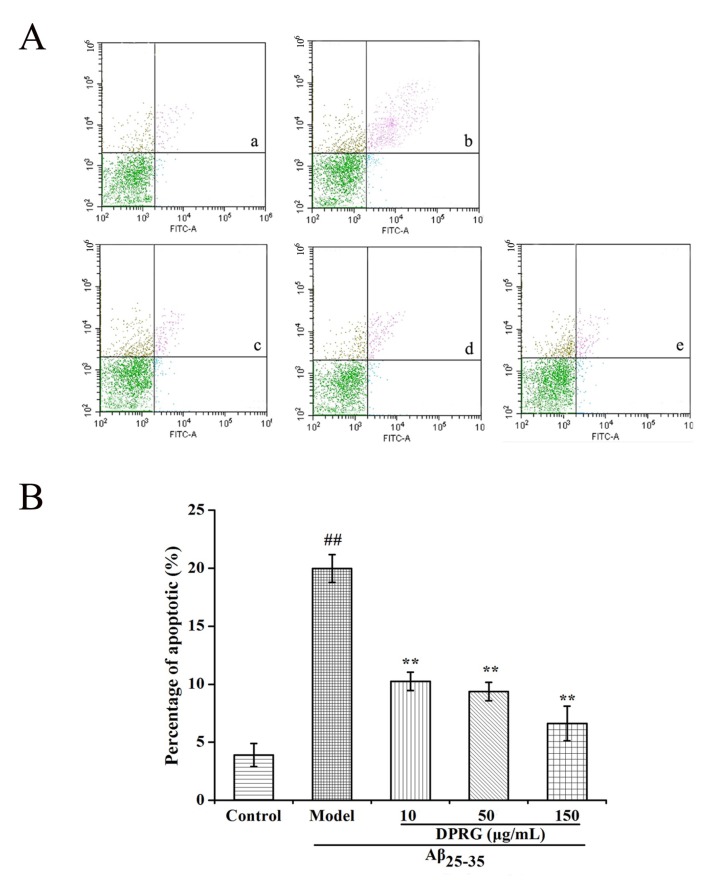
Effect of DPRG on apoptosis in PC12 cells induced by Aβ_25–35_. Cell apoptosis was identified by annexin-V/propidium iodide (PI) double-staining assay (**A**). PC12 cells were incubated in drug-free medium (control group: (a), 30 μM Aβ_25–35_ (model group: b), or pretreatment with 10 μg/mL (c), 50 μg/mL (d), and 150 μg/mL (e) of DPRG. Quantification analysis (**B**) are the means ± SD, *n* = 3. ^##^ *p* < 0.01 compared to control group; ** *p* < 0.01 compared with model group.

To examine whether the Aβ_25–35_-induced cell death of PC12 cells occurred by an apoptotic-like mechanism, flow cytometry analysis was utilized to detect the effect of DPRG on apoptosis in PC12 cells with an Annexin V-FITC/PI staining kit. In this method, Annexin V^+^ and PI^−^ cells were considered as early apoptotic cells, and the cells double-stained with both Annexin V and PI were defined as late apoptotic cells. Control cells were negative for both stains [29]. It is a well-established way to measure apoptosis in many experiments in vitro [31,32]. As shown in Figure 5, Aβ_25–35_ at 30 μM significantly increased both early and late apoptotic death in PC12 cells, with the total apoptosis rate reaching 20.0%. However, pretreatment with DPRG for 24 h, concentration-dependently decreased apoptosis compared to model group (*p* < 0.01). To our surprise, the percentage of apoptosis cell was decreased to only 6.63% at the high concentration of DPRG. The result suggested that DPRG protected PC12 cells from Aβ_25–35_ induced cytotoxicity by inhibiting apoptosis.

### 2.5. Effect of DPRG on Mitochondrial Dysfunction in Aβ_25–35_-treated PC12 Cells

**Figure 6:**
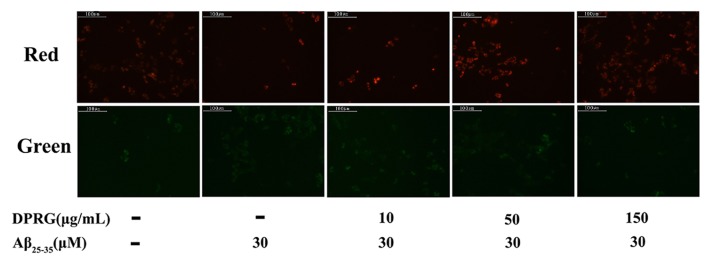
Effect of DPRG on Aβ_25–35_-treated PC12 cells by JC-1 staining. The figures from left to right were control group, model group, 10, 50, and 150 μg/mL DPRG pretreated groups.

MMP reduction, a central event of the apoptotic process, was usually assessed as a sensitive indicator of mitochondrial damage [33]. It has been demonstrated that Aβ aggregation could activate the mitochondrial apoptosis pathway and lead to a decrease in MMP [34]. To determine whether or not Aβ_25–35_-induced PC12 cell death associated with MMP, we used the mitochondrial probe JC-1 kit to measure MMP change in Aβ_25–35_-treated PC12 cells. In this study, we observed the cells after DPRG and Aβ_25–35_ treatment under the fluorescent microscope. The red and green fluorescence intensity in PC12 cells processed by JC-1 could represent the MMP change; the results are shown in Figure 6. The Aβ_25–35_-treated cells exhibited slight red fluorescence, while the cells in control group showed stronger red. After pretreatment with different concentrations of DPRG, the red florescence became stronger, especially in 50 and 150 μg/mL groups. The green fluorescence showed an opposite tendency compared with the fluorescence of red.

**Figure 7:**
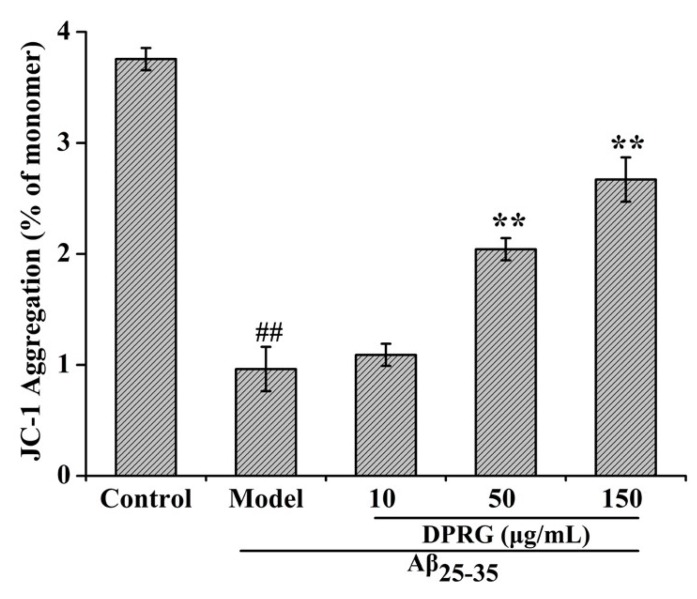
Quantification analysis are the means ± SD, *n* = 3. ^##^ *p* < 0.01, compared to control group, ** *p* < 0.01 compared to model group.

On the other hand, the MMP can be defined as the ratio of JC-1 aggregation (OD_590_) and monomer (OD_530_) [35]. A higher ratio of JC-1 aggregation and monomer means a higher MMP. As shown in Figure 7, exposure of PC12 cells to 30 μM Aβ_25–35_ for 24 h significantly decreased MMP in the PC12 cells of the control group. When the cells were pretreated with DPRG at the concentrations of 10, 50, and 150 μg/mL for 24 h, the ratio of OD_590_ and OD_530_ was increased at different levels, suggesting that the inhibition of DPRG on the apoptosis of PC12 cells was associated with mitochondrial membrane potential.

### 2.6. Effect of DPRG on Protein Expression in Aβ_25–35_-Treated PC12 Cells

**Figure 8:**
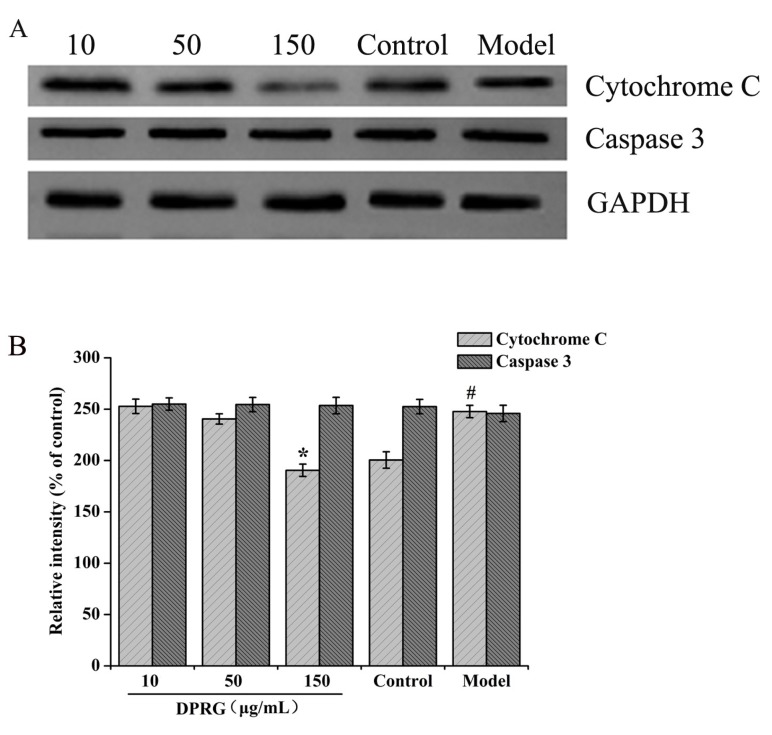
The related protein expression in Aβ_25–35_-treated PC12 cells. The protein expression of Cytc and caspase 3 in damaged cells (**A**). Quantification analysis (**B**) of the relative levels of Cytc and caspase 3 normalized to GAPDH. The values are presented as means ± S.D, *n* = 3. ^#^ *p* < 0.05, compared to control group; * *p* < 0.05 compared to model group.

As one of the main pathways, the mitochondrial pathway plays an important role in mediating apoptosis [36]. The release of Cytc from the mitochondria to the cytosol is pivotal in the apoptotic development [28]. Once released, the Cytc will activate the apoptosis-related proteins, including Bax, Bcl-2, as well as caspase 3, leading to neuronal apoptosis [37]. So we evaluated the protein expression of Cytc and caspase 3 in PC12 cells. As shown in Figure 8A,B, the results of Western blotting showed that the expression of Cytc protein in the model group increased compared with the control group. Compared with the model group, Cytc expression was significantly reduced in the 150 μg/mL DPRG group (*p* < 0.05) and showed no obvious reduction in both the 10 μg/mL DPRG and 50 μg/mL DPRG groups. And there were no changes of caspase 3 expression in the three DPRG groups. The results suggested that the inhibition of cell apoptosis was relative to Cytc decrease in PC12 cells induced by Aβ_25–35_.

## 3. Materials and Methods

### 3.1. Materials and Reagents

*Phellinus ribis* was collected in Jinan, Shandong province (China). DEAE-cellulose 23 was acquired from Serva (Heidelberg, Germany) and Superdex 30 was purchased from Amersham Biosciences (Uppsala, Sweden). Hydrogen peroxide (H_2_O_2_, 30%, *V*/*V*), NaNO_3_, and ascorbic acid (Vc) were purchased from Guoyao (Shanghai, China). Standard monosaccharides, including d-mannose (Man), d-xylose (Xyl), d-glucose (Glc), d-galactose (Gal), larabinose (Ara), l-rhamnose (Rha), and d-glucuronic acid (GlcU), were purchased from sigma (St. Louis, MO, USA). 3-(4, 5-Dimethylthiazol-2-yl)-2, 5-diphenyltetrazolium bromide (MTT) and DMSO were purchased from Sigma (St. Louis, MO, USA). Dulbecco’s modified Eagle’s medium (DMEM) and fetal bovine serum (FBS) were acquired from Gibco (Carlsbad, CA, USA). Aβ_25–35_ was purchased from Abcam. Annexin V-FITC kit was from Bestbio (Shanghai, China) and JC-1 kit was from Kaiji (Nanjing, China). The BCA protein kit, TBST buffer, rabbit polyclonal antibodies against Cytc, rabbit polyclonal antibodies against caspase 3, and mouse monoclonal against caspase 3 were from Biyuntian (Wuhan, China); anti-GAPDH was from Solarbio (Beijing, China). All other reagents used in the experiment were of analytically pure agent grade.

### 3.2. Preparation of PRG

The PRG was prepared according to Liu’s method with slight modifications [13]. In brief, dried crushed fruit bodies of *Phellinus ribis* were extracted with distilled water (1:6, *W*/*V*) at 95 °C for 5 h for three cycles. Then, the filtered extraction mixture was centrifuged at 3000 rpm for 10 min. The supernatant was concentrated at 50 °C under vacuum and precipitated by the addition of four volumes of ethanol at 4 °C overnight. Then, the precipitate was collected by centrifugation (4000 rpm, 10 min), dried at 50 °C, and a power was prepared. The power was dissolved in distilled water (1:20), and further precipitated by one volume of ethanol. After centrifugation, the supernatant was treated by adding three volumes of ethanol. The resulting precipitate was dried and dissolved in water and deproteinized by a combination of trypsin enzymolysis and Sevage method [38]. The polysaccharide obtained was dissolved in water, applied to DEAE-cellulose 23 column (4.5 cm × 30 cm), eluted by water and followed by 0.05 M NaCl. The fraction eluted with 0.05 M NaCl was dialyzed and further purified on a Superdex 30 gel column (1.6 cm × 80 cm) eluted with 0.05 M NaCl. Based on the colorimetric test for total carbohydrate by the phenol–sulfuric acid method, the main fraction was obtained, dialyzed, lyophilized, and a white power (named PRG) was obtained.

### 3.3. Preparation of DPRG

The degradation of PRG was conducted using a H_2_O_2_-Vc method [11]. Briefly, PRG (20 mg) was dissolved in 10 mL distilled water and different concentrations of H_2_O_2_ and Vc (molar ratio was 1:1) were added into the solution. Then hydrolysis of PRG was carried for the designated reaction temperatures (40 °C, 50 °C, and 60 °C) and times (1 h, 1.5 h, and 2 h). After the reaction, the mixture was dialyzed for 3 days, and lyophilized to obtain the degraded PRG. The degraded PRG was sequentially purified through Sephacryl S-100; the main fraction was collected and lyophilized to obtain DPRG (0.32 g). As determined by a phenol–sulfuric acid colorimetric method using glucose as a standard, the total sugar content of DPRG was 97.7%.

### 3.4. Experimental Design of RSM

A three-factor design, which was used for the optimization process with the Design Expert Software (V 8.0.5, Stat-Ease, USA), was statistically applied to examine the effects of independent variables at three levels on the response dependent variable (named cell viability of PC12 cells induced by Aβ_25–35_, conducted as “2.9”) to allow optimization of PRG degradation. Three independent variables, concentration of H_2_O_2_-Vc A, degradation temperature B, and degradation time C, were selected for the study. The Box–Behnken design (BBD) is shown in Table 2. The experimental design consisting of 17 sets of experimental points carried out in random order are presented in Table 1. Under the optimal conditions, PRG was degraded and sequentially purified through Sephacryl S-100, the main fraction was collected and lyophilized to get the degraded PRG (DPRG).

### 3.5. Molecular Weight Analysis

The molecular weight of DPRG was measured using high-performance gel permeation chromatography (HPGPC, Water Breeze, Waters, Milford, MA, USA) on a Agilent 1200 system (Agilent Technologies, Palo Alto, CA, USA) coupled to a refractive index detector (RID, Agilent Technologies, Palo Alto, CA, USA). The mobile phase was 0.1 M NaNO_3_, and the sample was dissolved in mobile phase and passed through a 0.45 μm filter before use. The injection volume was 10 μL and at a flow rate of 1.0 mL/min. The temperature of the column was maintained at 40 °C. The molecular weight of DPRG was conducted using dextran standards with different molecular weights.

### 3.6. Characterization of DPRG

The total carbohydrate content of DPRG was determined by a phenol–sulfuric acid colorimetric method using glucose as a standard. The Fourier-transform infrared (FTIR, Thermo-Electron, Madison, WI, USA) spectra (KBr pellets) was measured on a PerkinElmer Frontier. The uronic acids of the DPRG were identified by thin layer chromatography (TLC) on a plate (10 cm × 20 cm) precoated with the slurry made of silica gel (6 g), NaH_2_PO_4_·2H_2_O (0.75 g) and 0.5% CMC-Na (16 mL). The developing agent was a mixture of acetic ether, pyridine, water, and acetic acid (5:5:3:1). The sugars were identified by aniline–diphenylamine–phosphoric acid reagent (the ratio of 2% aniline acetone solution, 2% diphenylamine acetone solution, and 85% phosphoric acid is 5:5:1) onto the plate and heating at 105 °C for 10 min. The monosaccharide composition of DPRG was analyzed using an Agilent HP6890 GC with a FID injector (Agilent Technologies, USA) equipped with a HP-5 capillary column (30 m × 0.25 mm) and a flame ionization detector. The temperature program was from 170 °C to 230 °C with a rate of 2 °C/min and at 230 °C for 5 min [39,40,41].

### 3.7. Cell Culture and Treatment

PC12 cells were obtained from the Department of Pharmacology, Shandong University. The cells were cultured in DMEM supplemented with 10% FBS (*V*/*V*) and penicillin (100 IU/mL) at 37 °C in a humidified atmosphere containing 5% CO_2_ [42]. The culture media was replaced every other day and passaged every two days.

### 3.8. Preparation of Aβ_25–35_

Aβ_25–35_ 1 mg (Abcam, Cambridge, MA, USA), was dissolved in deionized distilled water (1 mL) and incubated at 37 °C for a week to induce its aggregation. After 7 days, the solution was stored at −20 °C and diluted to the desired concentrations by adding cell culture medium before using it [10].

### 3.9. Cell Viability Assay

Cell viability was evaluated by MTT assay [43,44,45]. Briefly, PC12 cells (8 × 10^3^ cells/well) were seeded into 96-well plates and grown to confluence. Then, the cells were divided into five groups randomly: Group 1 (control group): treatment with medium containing 2% serum; Group 2 (model group): treatment with medium containing 2% serum; Group 3: treatment with 10 μg/mL DPRG; Group 4: treatment with 50 μg/mL DPRG; Group 5: treatment with 150 μg/mL DPRG. Twenty-four hours later, 30 μM Aβ_25–35_ was added except wells in control group for another 24 h. After incubation, the cells were treated with 5 mg/mL MTT for 4 h at 37 °C and then the medium was carefully removed. One-hundred microliters of DMSO was added to dissolve the formazan crystals, and the formazan absorbance was measured at 570 nm on a microplate reader (Bio Tek Instruments). The cell viability calculation formula was as follows (Equation (2)):

Cell viability% = (OD_treatment group_)/(OD_control group_) × 100% (2)

### 3.10. Flow Cytometric Analysis

Apoptosis was detected using the Annexin V-FITC/PI double staining kit according to manufacturer’s instruction. Briefly, after DPRG and Aβ_25–35_ treatment which was similar with “3.9”, cells were collected at 2000 rpm for 5 min, washed with ice-cold PBS, and then resuspended in 400 μL binding buffer. Five microliters of Annexin V-FITC and 10 μL PI were added into cells, after which they were incubated in the dark at room temperature for 15 min. Quantitative analysis of the level of apoptosis was measured with a flow cytometer (Beckman, Germany). Apoptotic cells were analyzed as a percentage of the total number of cells in control group.

### 3.11. MMP Measurement

A JC-1 Mitochondrial Membrane Potential Detection Kit (Kaiji, Jiangsu) was used to measure the loss of MMP in PC12 cells. After treatment, cells were incubated at 37 °C for 15 min in the dark with JC-1. After incubation, cells were centrifugated at 2000 rpm for 5 min, washed two times with 1× Incubation Buffer, and then cells were resuspended in 0.5 mL Incubation Buffer and detected by the absorbance at 590 nm (red, high membrane potential, JC-1 aggregation) and 530 nm (green, low membrane potential, JC-1 monomer). MMP was expressed as the ratio of JC-1 aggregation and JC-1 monomer. In addition, the fluorescence intensity was also detected using fluorescent microscope (Olympus, Tokyo, Japan).

### 3.12. Western Blotting

After treatment of DPRG and Aβ_25–35_ according to Section 3.9, the cells from different groups were homogenized with 200 μL of ice-cold RIPA lysis buffer on ice for 30 min. The homogenate was rotated at 4 °C for 20 min and centrifuged at 12,000 rpm at 4 °C for 30 min and the supernatant was collected. Protein concentrations were determined using the Enhanced BCA Protein kit. Equal amounts of samples were separated by 10% SDS-PAGE and transferred to Polyvinylidene diuoride (PVDF) membranes. After blocking with 5% nonfat milk for 2 h at room temperature, the membranes were washed with TBST buffer three times (10 min/each time) and incubated with anti-Cytc and anti-caspase 3 antibody at 4 °C overnight. Subsequently, the membranes were washed and incubated with HRP-conjugated secondary antibody at room temperature for 1h. The proteins were then detected.

### 3.13. Statistical Analysis

All data obtained were expressed as mean ± standard deviation (SD). The statistical significance of different groups was determined using one-way of variance (ANOVA). All statistical analysis was performed with the SPSS 17.0 software, * *p* < 0.05 was considered as statistical significance.

## 4. Conclusions

At present, more and more evidence indicates that natural polysaccharides extracted from animals and plants possess a range of biological activities [11,12]. In the previous work, a polysaccharide named PRG was isolated from the medicinal fungus *Phellinus ribis*. PRG significantly promoted the neurite outgrowth of the nerve growth factor-mediated PC12 cells, exhibiting the neurotrophic activity [13]. However, the biological activities of the polysaccharide are usually limited by the high molecular weight [14]. It is necessary to get active structural unit of polysaccharide with lower molecular weight to increase water solubility or accelerate absorption and transport. Therefore, the present paper is concerned with the optimization of the degradation of PRG conditions by RSM. The statistical analysis showed that the optimum conditions were found to be the concentration of H_2_O_2_-Vc 17 mM, degradation temperature 50 °C, degradation time 1.6 h, respectively. Under these conditions, the active structural unit of PRG with lower molecular weight was obtained. Its experimental PC12 cells viability was 83.4 ± 0.15%, which was very close to the predicted cell viability of 83.5%.

Alzheimer’s disease (AD) is a deadly chronic neurodegenerative disorders in the world, many studies reported that one of the causes of AD was the accumulation of amyloid-β (Aβ) fibrillar deposition [4]. Aβ_25–35_ could induce a direct toxic effect on the neurons, leading to apoptosis of neurons. This paper studied the protective mechanism of DPRG on Aβ_25–35_-induced damage in PC12 cells. The results demonstrated that the inhibition of DPRG on the apoptosis of PC12 cells was associated with MMP and Cytc.

We found that both DPRG and PRG showed protective effects on Aβ_25–35_-induced damage in PC12 cells, but due to the larger molecular weight and poor water solubility of PRG, further application of PRG was limited. Therefore, we further studied the protective mechanism of DPRG on Aβ_25–35_-induced damage in PC12 cells. In this paper, we mainly studied the mitochondrial apoptosis pathway and we found that DPRG could inhibit apoptosis by increasing MMP levels and decreasing Cytc protein expression. In fact, the apoptosis pathways include the endoplasmic reticulum pathway, the death receptor pathway, etc. We will further study the impact of DPRG on these pathways.

Although some achievements have been made in the prevention and treatment of AD, there is still no evidence that one drug can terminate or reverse the progression of AD. The treatment level is only to improve symptoms or delay progression. Therefore, in the present paper, the inhibition of Aβ-induced neuronal apoptosis may provide a promising approach for the prevention and treatment of neurodegenerative diseases especially AD.

## Figures and Tables

**Table 1:** Box–Behnken designs and results.

No.	A	B	C	Y
1	0	−1	1	74.8
2	0	1	1	77.0
3	0	−1	−1	73.3
4	1	0	−1	77.4
5	−1	−1	0	70.3
6	−1	0	1	72.9
7	−1	1	0	72.8
8	1	0	1	79.3
9	1	−1	0	77.1
10	0	1	−1	74.8
11	1	1	0	78.6
12	−1	0	−1	71.6
13	0	0	0	83.1
14	0	0	0	82.9
15	0	0	0	82.5
16	0	0	0	82.8
17	0	0	0	82.4

**Table 2:** Experiments design for response surface methodology (RSM).

Variables	Coded Symbols	Coded Levels		
		−1	0	1
Concentration (mM)	A	10	15	20
Temperature (°C)	B	40	50	60
Time (h)	C	1	1.5	2

**Table 3:** Analysis of variance for regression model.

Source	Sum of Squares	df	Mean Square	*F*-Value	*p*-Value Prob > F	
Model	304.16	9	33.80	617.18	<0.0001	Significant
A	75.89	1	75.89	1385.96	<0.0001	***
B	7.37	1	7.37	134.65	<0.0001	***
C	5.81	1	5.81	106.18	<0.0001	***
AB	0.26	1	0.26	4.66	0.0678	
AC	0.13	1	0.13	2.30	0.1730	
BC	0.12	1	0.12	2.17	0.1839	
A^2^	63.67	1	63.67	1162.83	<0.0001	***
B^2^	74.05	1	74.05	1352.39	<0.0001	***
C^2^	54.38	1	54.38	993.10	<0.0001	***
Residual	0.38	7	0.055			
Lack of Fit	0.057	3	0.019	0.23	0.8704	not significant
Pure Error	0.33	4	0.082			
Cor Total	304.54	16				

R^2^ = 0.9987; R^2^_Adj_ = 0.9971; C.V.% = 0.30%; *** *p* < 0.001 extremely significant.

## References

[1] Diana F., Javier D., Lidia A. (2018). A Study of Amyloid-β and Phosphotau in Plaques and Neurons in the Hippocampus of Alzheimer’s Disease Patients. J. Alzheimers Dis..

[2] Yang S., Zhang R., Wang G., Zhang Y. (2017). The development prospection of HDAC inhibitors as a potential therapeutic direction in Alzheimer’s disease. Transl. Neurodegener..

[3] Panahi Y., Mohammadhosseini M., Abadi A., Akbarzadeh A., Mellatyar H. (2016). An Update on Biomedical Application of Nanotechnology for Alzheimer’s Disease Diagnosis and Therapy. Drug Res..

[4] Yamakawa M., Uchino K., Watanabe Y., Adachi T., Nakanishi M., Ichino H., Hongo K., Mizobata T., Kobayashi S., Nakashima K. (2016). Anthocyanin suppresses the toxicity of Aβ deposits through diversion of molecular forms in in vitro and in vivo models of Alzheimer’s disease. Nutr. Neurosci..

[5] Li Y., Guan S., Liu C., Chen X., Zhu Y., Xie Y., Wang J., Ji X., Li L., Li Z. (2018). Neuroprotective effects of Coptis chinensis Franch polysaccharide on amyloid-beta (Aβ)-induced toxicity in a transgenic Caenorhabditis elegans model of Alzheimer’s disease (AD). Int. J. Biol. Macromol..

[6] Bag S., Chaudhury S., Pramanik D., DasGupta S., Dasgupta S. (2016). Hydrophobic tail length plays a pivotal role in amyloid beta (25-35) fibril-surfactant interactions. Proteins.

[7] Caruso G., Fresta C.G., Lazzarino G., Distefano D.A., Parlascino P., Lazzarino G., Caraci F. (2018). Sub-Toxic Human Amylin Fragment Concentrations Promote the Survival and Proliferation of SH-SY5Y Cells via the Release of VEGF and HspB5 from Endothelial RBE4 Cells. Int. J. Mol. Sci..

[8] Zimbone S., Monaco I., Gianì F., Pandini G., Copani A.G., Giuffrida M.L., Rizzarelli E. (2018). Amyloid Beta monomers regulate cyclic adenosine monophosphate response element binding protein functions by activating type-1 insulin-like growth factor receptors in neuronal cells. Aging Cell.

[9] Caruso G., Distefano D.A., Parlascino P., Fresta C.G., Lazzarino G., Lunte S.M., Nicoletti V.G. (2017). Receptor-mediated toxicity of human amylin fragment aggregated by short- and long-term incubations with copper ions. Mol. Cell. Biochem..

[10] Ling J. (2018). Radix Hedysari Polysaccharide Protects PC12 Cells against Aβ_25–35_-Induced Apoptosis via PRKCB/ERK-Dependent Pathways.

[11] Chen B., Shi M., Cui S., Hao S., Hider R., Zhou T. (2016). Improved antioxidant and anti-tyrosinase activity of polysaccharide from Sargassum fusiforme by degradation. Int. J. Biol. Macromol..

[12] Lemieszek M., Nunes F., Cardoso C., Marques G., Rzeski W. (2018). Neuroprotective properties of, Cantharellus cibarius, polysaccharide fractions in different, in vitro, models of neurodegeneration. Carbohydr. Polym..

[13] Liu Y., Liu C., Jiang H., Zhou H., Li P., Wang F. (2015). Isolation, structural characterization and neurotrophic activity of a polysaccharide from *Phellinus ribis*. Carbohydr. Polym..

[14] Zhao X., Li B., Xue C., Sun L. (2012). Effect of molecular weight on the antioxidant property of low molecular weight alginate from *Laminaria japonica*. J. Appl. Phycol..

[15] Zhao Y., Shi Y., Yang H., Mao L. (2016). Extraction of Angelica sinensis polysaccharides using Ultrasound-assisted way and its bioactivity. Int. J. Biol. Macromol..

[16] Pan P., Jin W., Li X., Chen Y., Jiang J., Wan H., Yu D. (2018). Optimization of multiplex quantitative polymerase chain reaction based on response surface methodology and an artificial neural network-genetic algorithm approach. PLoS ONE.

[17] Gu F., Xu F., Tan L., Wu H., Chu Z., Wang Q. (2012). Optimization of Enzymatic Process for Vanillin Extraction Using Response Surface Methodology. Molecules.

[18] Wang R., Chen P., Jia F., Tang J., Ma F. (2012). Optimization of polysaccharides from *Panax japonicus* C.A. Meyer by RSM and its anti-oxidant activity. Int. J. Biol. Macromol..

[19] González-Centeno M., Knoerzer K., Sabarez H., Simal S., Rosselló C., Femenia A. (2014). Effect of acoustic frequency and power density on the aqueous ultrasonic-assisted extraction of grape pomace (*Vitis vinifera* L.)—A response surface approach. Ultrason Sonochem..

[20] Mohammad A., Abdulhameed A., Jawad A. (2019). Box-Behnken design to optimize the synthesis of new crosslinked chitosan-glyoxal/TiO_2_ nanocomposite: Methyl orange adsorption and mechanism studies. Int. J. Biol. Macromol..

[21] Gupta P., Nayak K. (2016). Optimization of keratin/alginate scaffold using RSM and its characterization for tissue engineering. Int. J. Biol. Macromol..

[22] Maran J.P., Mekala V., Manikandan S. (2013). Modeling and optimization of ultrasound-assisted extraction of polysaccharide from *Cucurbita moschata*. Carbohydr. Polym..

[23] Gu P., Xu S., Zhou S., Liu Z., Sun Y., Ou N., Hu Y., Liu J., Wu Y., Wang X. (2018). Optimization of angelica sinensis polysaccharide-loaded Poly (lactic-co-glycolicacid) nanoparticles by RSM and its immunological activity in vitro. Int. J. Biol. Macromol..

[24] Kuo C., Hsiao F., Chen J., Hsieh C., Liu Y., Shieh C. (2013). Kinetic aspects of ultrasound-accelerated lipase catalyzed acetylation and optimal synthesis of 4′-acetoxyresveratrol. Ultrason Sonochem..

[25] Shan H., Chu Y., Chang P., Yang L., Wang Y., Zhu S., Zhang M., Tao L. (2017). Neuroprotective effects of hydrogen sulfide on sodium azideinduced autophagic cell death in PC12 cells. Mol. Med. Rep..

[26] Sheng Y., Liu G., Wang M., Lv Z., Du P. (2017). A selenium polysaccharide from, *Platycodon grandiflorum*, rescues PC12 cell death caused by H_2_O_2_, via inhibiting oxidative stress. Int. J. Biol. Macromol..

[27] Hao L., Zhang Q., Yu T., Cheng Y., Ji S. (2011). Antagonistic effects of ultra-low-molecular-weight heparin on Aβ_25–35_-induced apoptosis in cultured rat cortical neurons. Brain Res..

[28] Zhang H., Cao Y., Chen L., Wang J., Tian Q., Wang N., Liu Z., Li J., Wang N., Wang X. (2015). A polysaccharide from *Polygonatum sibiricum* attenuates amyloid-β-induced neurotoxicity in PC12 cells. Carbohydr. Polym..

[29] Zhang Q., Li J., Liu C., Song C., Li P., Yin F., Xiao Y., Li J., Jiang W., Zong A. (2015). Protective effects of low molecular weight chondroitin sulfate on amyloid beta (Aβ)-induced damage in vitro and in vivo. Neuroscience.

[30] Van Tonder A., Joubert A., Cromarty A. (2015). Limitations of the 3-(4,5-dimethylthiazol-2-yl)-2, 5-diphenyl-2H-tetrazolium bromide (MTT) assay when compared to three commonly used cell enumeration assays. BMC Res. Notes.

[31] Zhou X., Liu Z., Long T., Zhou L., Bao Y. (2018). Immunomodulatory effects of herbal formula of astragalus polysaccharide (APS) and polysaccharopeptide (PSP) in mice with lung cancer. Int. J. Biol. Macromol..

[32] Zapotocky M., Mejstrikova E., Smetana K., Stary J., Trka J., Starkova J. (2012). Valproic acid triggers differentiation and apoptosis in AML1/ETO-positive leukemic cells specifically. Cancer Lett..

[33] Menges S., Minakaki G., Schaefer P., Meixner H., Prots I., Schlötzer-Schrehardt U., Friedland K., Winner B., Outeiro T., Winklhofer K. (2017). Alpha-synuclein prevents the formation of spherical mitochondria and apoptosis under oxidative stress. Sci. Rep..

[34] Cheng W., Chen W., Wang P. (2018). Asiatic acid protects differentiated PC12 cell from Aβ-induced apoptosis and tau hyperphosphorylation via regulating PI3K/Akt/GSK-3β signaling. Life Sci..

[35] Wang J., Zhou T., Wang T., Wang B. (2018). Suppression of lncRNA-ATB prevents amyloid-β-induced neurotoxicity in PC12 cells via regulating miR-200/ZNF217 axis. Biomed. Pharmacother..

[36] Hackenbeck T., Huber R., Schietke R., Knaup K., Monti J., Wu X., Klanke B., Frey B., Gaipl U., Wullich B. (2011). The GTPase RAB20 is a HIF target with mitochondrial localization mediating apoptosis in hypoxia. Biochim. Biophys. Acta.

[37] Xiong S., Mu T., Wang G., Jiang X. (2014). Mitochondria-mediated apoptosis in mammals. Protein Cell.

[38] Wang L., Duan Y., Ma Y., Ding H., Li E. (2008). Studies on extraction and antioxidant function of polysaccharides from *Sophora japonica*. J. Northwest A F Nat. Sci. Ed..

[39] Ru Y., Chen X., Xu J., Huang L., Jiang M., Guo L., Lin Z., Qiu B., Wong K. (2018). Hypoglycemic effects of a polysaccharide from *Tetrastigma hemsleyanum* Diels & Gilg in alloxan-induced diabetic mice. Chem. Biodivers..

[40] Fujii M., Sato Y., Ito H., Masago Y., Omura T. (2012). Monosaccharide composition of the outer membrane lipopolysaccharide and O-chain from the freshwater cyanobacterium *Microcystis aeruginosa* NIES-87. J. Appl. Microbiol..

[41] Cao C., Huang Q., Zhang B., Li C., Fu X. (2018). Physicochemical characterization and in vitro hypoglycemic activities of polysaccharides from *Sargassum pallidum* by microwave-assisted aqueous two-phase extraction. Int. J. Biol. Macromol..

[42] Xu J., Zhou L., Weng Q., Xiao L., Li Q. (2019). Curcumin analogues attenuate Aβ-induced oxidative stress in PC12 cells via Keap1/Nrf2/HO-1 signaling pathways. Chem. Biol. Interact..

[43] Deng Q., Yang X. (2014). Protective effects of *Gynostemma pentaphyllum* polysaccharides on PC12 cells impaired by MPP^+^. Int. J. Biol. Macromol..

[44] Lee S., Youn K., Jeong W., Ho C., Jun M. (2017). Protective Effects of Red Ginseng Oil against Aβ_25–35_-Induced Neuronal Apoptosis and Inflammation in PC12 Cells. Int. J. Mol. Sci..

[45] Silakhori S., Hosseinzadeh H., Behbahani F.S., Mehri S. (2019). Neuroprotective effect of clavulanic acid on trimethyltin (TMT)-induced cytotoxicity in PC12 cells. Drug Chem. Toxicol..

